# The Comparison of the Effect of Three Anesthetic Induction Regimens on the Arterial Oxygen Saturation in Children with Tetralogy of Fallot Undergoing Cardiac Surgery

**Published:** 2011-10-01

**Authors:** A R Tavakollian, E Allahyary

**Affiliations:** 1 Anesthesiology and Critical Care Research Center, Shiraz University of Medical Sciences, Shiraz, Iran

**Keywords:** Tetralogy of fallot, SaO2%, Halothane, Ketamine

## Abstract

**Background:**

Tetralogy of fallot (TOF) is the most common cyanotic congenital heart disease. Anesthesia induction is a challenging issue in these patients due to the risk of worsening hypoxemia following decrease in pulmonary blood flow. We evaluated the effect of three anesthetic induction regimens on the arterial oxygen saturation (SaO2%) in children with TOF.

**Methods:**

Seventy six children aged 50 days to 15 years old with TOF, scheduled in Nemazee and Faghihi hospitals to undergo elective cardiac surgery during 1385-1388 were randomly divided into 3 groups to receive three anesthetic induction agents including ketamine (2 mg/kg, IV), ketamine (5 mg/kg, IM) and halothane for gas induction. SaO2% and heart rate were recorded before induction and thereafter every 1 minute during induction of anesthesia till 10 min post-induction.

**Results:**

There were not significant differences between three groups regarding pattern of changes in SaO2% during 10 min post-induction. All three groups showed an increase in SaO2% committed over 6th minute but this pattern was not seen after that time. In addition, there were not significant differences among groups according to heart rate in the study period.

**Conclusion:**

It seems that anesthesia induction in TOF patients with ketamine IV and IM and halothane did not have significant adverse effects on SaO2%. Indeed, oxygenation during induction may offset other possible adverse effects of induction drugs on SaO2%.

## Introduction

Tetralogy of fallot (TOF) is the most common cyanotic congenital heart disease (CCHD) which characterized by a large single ventricular septal defect (VSD), an overridden aorta, obstruction to right ventricular out flow and right ventricular hypertrophy. Hypercyanotic attacks in TOF patients are common and characterized by sudden spells of hypoxemia due to a sudden decrease in pulmonary blood flow (PBF). Surgical interventions in these patients include total correction and some palliative procedures to increase PBF transiently.[1]

Anesthesia management in these patients require understanding of the events or drugs that influence the magnitude of right to left (Rt to Lt) shunting of blood, right ventricular outflow obstruction (affected by myocardial contractility) and resulting PBF and arterial oxygen saturation (SaO2%). Both intravenous (IV) and inhalational anesthetic regimens have been recommended for induction of anesthesia in patients with TOF. Ketamine is the suggested anesthetic drug for induction due to its beneficial cardiovascular effect on increasing systemic vascular resistance (SVR) and resulting decreased Rt to Lt shunting via VSD and improved oxygenation due to increased PBF. On the other hand, ketamine can cause infundibular cardiac muscle spasm via its positive inotropic effect. Anesthetic induction with volatile agents such as sevoflurane and halothane are acceptable but have some adverse and/or beneficial effects on PBF and SaO2%.[2]

Therefore in the present study, we designed a way to compare SaO2% during anesthesia induction with three different regimens of anesthetic induction consisting of IV ketamine, ketamine intramuscularly (IM) and gas induction with halothane in infants and children with TOF anomaly underwent cardiac surgery.

## Materials and Methods

After approval from the institutional review board, ethical committee and getting parental consent, 76 infants and children aged 50 days to 15 years old with diagnosis of TOF and positive history of hypercyanotic spell presenting for elective cardiac surgery in Nemazee and Faghihi hospitals in Shiraz, southern Iran affiliated to Shiraz University of Medical Sciences were enrolled during 2007 -2010. Patients with history of previous palliative cardiac surgery were excluded from the study because they were unlikely to have a hypercyanotic spells. Also patients with any lung disease were excluded. Infants less than 9 months and more than 8 y/o received no premedication and others received midazolam (0.3 mg/kg, maximum 15 mg/kg, orally) 30 min prior to induction of anesthesia under close observation.

**Fig. 1:**
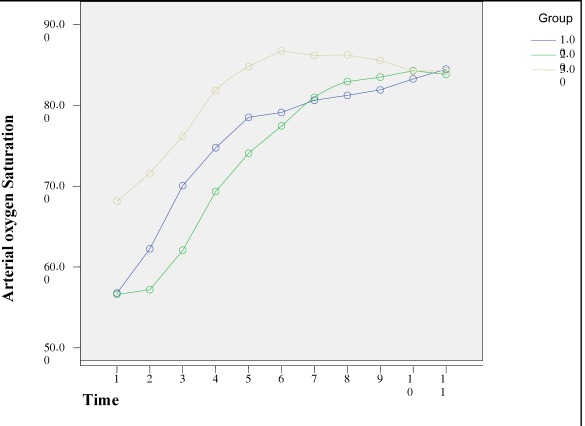
Pattern of changes in arterial oxygen saturation during study period in the three groups. Group 1: IV ketamine, group 2: IM ketamine, group 3: Halothane.

On arrival in the operating room, the patients were placed supine and monitored with a precordial stethoscope, ECG, non invasive blood pressure and finger probe pulse oximeter (Nellcor Inc, Hayward, CA). In all cases, pulse oximeter probe was placed to the right thumb. While patients breathing room air, a set of baseline data were recorded. The patients were randomized to one of the three study groups for receiving ketamine (2 mg/kg, IV) in 20 cases (KIV group), ketamine (5 mg/kg, IM) in 26 patients (KIM group) and gas induction with halothane in 25 cases which was incrementally raised from 0.5 to 4% concentration via a face mask with increasing rate of 0.5% concentration each 2-3 breath and then declined to 2% immediately after loss of eyelash reflex (H group). All patients were oxygenated with oxygen (6-7 L/min) before and during induction via an appropriate sized face mask. An intravenous catheter was placed in the ward before arriving to the operating room in each patient. Oral endotracheal intubation was performed following administration of fentany (l2 µg/kg, IV) and pancuronium (0.1 mg/kg, IV) and then an intra-arterial line and a central venous line was inserted for all cases. In all children, heart rate (HR) and SaO2% were recorded at baseline in awaken state and then every 1 min during induction for 10 minutes by a person who was blinded to study groups and was not involved in anesthesia management. SaO2% determined by the pulse oximeter was compared with oxygen saturation measured directly by arterial blood gas analysis provided by an arterial blood sample from the arterial line at 2 time points of operation in all patients (Figure 1).

Collected data were analyzed by SPSS software (Version 16, Chicago, IL, USA). Data were presented as mean±SD (standard deviation) for quantitative variables and frequency for qualitative ones. Age, weight, baseline HR and SaO2% between groups were compared by Kruskal-Wallis test. ANOVA with repeated measures was used to compare SaO2% time course between groups. Sphericity assumption was not satisfied, and then adjusted p value given by Greenhouse-Geisser was used. Statistical significance was assumed to p<0.05.

## Results

Seventy six patients of the three groups completed the study. There were not significant differences in age (p=0.140), weight (p=0.072), baseline SaO2% (p=0.205) and HR (p=0.164) among groups (Table 1). In all groups, SaO2% increased significantly during the study time in comparison to awaken measurements when patients breathed room air. ANOVA with repeated measures revealed that there were no differences in the mean of SaO2% over ten minutes between three groups (F=1.62, df= 2, p= 0.2).

Figure 1 shows that there was a time trend effect. All three groups showed increase of SaO2% committed over six minutes without significant differences in SaO2% between T1 versus T2 (p=0.039), T2 versus T3 (p<0.001), T3 versus T4 (p=0.000<0.001), T4 versus T5 (p=0.001) and T5 versus T6 (p=0.017). But this pattern was not seen after that time, in a way as p=0.254, 0.138, 0.766, 0.617 and 0.464 between T6 and T7, T7 and T8, T8 and T9, T9 and T10, T10 and T11 respectively. There was no time group interaction effect (F=2.02, df=7.1, p=0.058) and all groups had the same time trend over ten minutes.

Evaluation and comparison of HR between groups showed that there were not significant differences among groups (p=0.160) during the 10 minutes of the study period.

**Table 1:** Demographic data and baseline arterial oxygen saturation and heart rate in study patients

	**Halothane**	**IM Ketamine**	**IV Ketamine**
Age (months)	53.6±44.4	55.8±36.4	57.2±40.4
Weight (kg)	13.7±5.9	13.9±4.1	14.7±8.8
Baseline HR a	140±29	132.8±21.8	125.5±24.2
Baseline SaO2	% 75.7±2.7	73.8±2.6	81.4±3.4

^a^ HR: heart rate, SaO2%: arterial oxygen saturation

## Discussion

TOF was initially described in the 19th century as an association of four anatomic findings: VSD, subpulmonary stenosis, aortic override of the ventricular septum, and right ventricular (RV) hypertrophy. Patients with minimal RV outlet obstruction have unrestricted PBF and a left to right shunt through the VSD. Conversely, patients with severe obstruction would be cyanosed with saturation in the 70% to 80% range preoperatively as a result of right to left shunting. RV outlet obstruction is often dynamic and may cause profound cyanosis (hypercyanotic spells). Management of anesthesia in TOF patients requires a thorough understanding of those events and drugs can alter the magnitude, of the Rt to Lt shunt. For example, when shunt magnitude is acutely increased, there are associated decreases in PBF and SaO2%.

The magnitude of Rt to Lt shunt can be increased by (i) Decreased systemic vascular resistance (SVR), (ii) Increased pulmonary vascular resistance (PR), and (iii) Increased myocardial contractility which attenuates infandibular obstruction to eject of blood by the RV. Published reports are inconclusive and controversial with regards to the effect of various anesthetic agents on PBF and SaO2% in cyanotic congenital heart disease. The optimal induction regimen for patients with congenital heart disease should maintain cardiovascular stability.

Ketamine is the recommended anesthetic agent in patients with TOF, nevertheless; the use of ketamine remains controversial because it increases pulmonary arterial pressure in adult patients.[3][4] A small number of studies evaluating the effect of ketamine on pulmonary artery pressure in children showed conflicting results.[5][6][7][8] Some studies reported that ketamine did not alter HR, blood pressure or intracardiac shunt in cyanotic congenital heart disease either prior to surgical repair or after surgical repair.[7][8] Another study also showed that ketamine did not increase PR in children with pulmonary hypertension undergoing sevoflurane anesthesia and spontaneous ventilation.[9]

The adverse effect of ketamine on PBF (increased PR) may be opposed by its increasing effect on SVR and decreased magnitude of Rt to Lt shunt. Animal investigations suggest that the effects of ketamine on PR are complex. It increased concentrations of epinephrine and norepinephrine in plasma and increased PR in isolated rat lungs.[10][11] However, later work with several animal models reported that ketamine caused direct pulmonary artery vasodilatation. This effect is endothelium independent and mediated by a reduction of calcium in vascular smooth muscle cells by inhibitions of both voltage-gated calcium influx and norepinephrine induced calcium release from intracellular stores.[4][5][6][7][8][9][10][11][12][13][14][15][16] Ketamine has also been noted to attenuate endothelium-dependent pulmonary vasorelaxation in response to acetylcholine and bradykinin by inhibiting both the nitric oxide and the endothelium derived hyperpolarized factor components of the response. [17][18] Finally, animal data shows ketamine has the potential to increase or inhibit pulmonary vasodilatation and the clinical effect most likely depends on the integration of multiple mechanisms.[9]

In contrast, positive inotropic effect of ketamine on myocardium may cause infundibular spasm of cardiac muscle and following increasing Rt to Lt shunt magnitude, decreased PBF and SaO2%. Intravenous and intramuscular injections are acceptable routes of administration of ketamine in anesthesia induction. It seems that adverse or beneficial hemodynamic effects of IM ketamine appear more gradual than IV ketamine. We compare these two routes of administration of ketamine in TOF patients regarding effect on SaO2% and HR and observed that both groups of ketamine (IV and IM) had similar increasing course in SaO2% in comparison to awaken values during the first 10 min after induction of anesthesia.

Gas induction with volatile agents is another acceptable anesthetic induction regimen in TOF patients. We found the similar increasing course of SaO2% in TOF children anesthetized with halothane as gas induction in comparison to ketamine groups despite its significant vasodilatory effect. A large decrease in SVR would be expected to result in a net increase in Rt to Lt shunting and aggravated hypoxemia. However, decrease in SaO2% was not noticed in any of the patients in halothane group. This finding was also reported by William and co-workers when compared the effect of halothane and IM ketamine on SaO2% in children with cyanotic congenital heart disease.[19]

There are some explanations for this observation. In our patients, tissue oxygen utilization decreased as the children progressed from awaken premedicated state to the anesthetized situation. This resulted in both a decrease in PBF requirement and an increase in mixed venous oxygenation. Keeping the PBF constant, a rise in mixed venous oxygenation has been shown to increase SaO2% in cyanotic congenital heart disease.[20] Another reason may be a decrease in RV outlet obstruction due to myocardial depressant effect of halothane resulting increase in PBF and SaO2%.[21] Finally, halothane induced hypotension that might have been due to decreased cardiac output rather than a decrease in SVR. Under these conditions, PBF was sufficient to increase SaO2%. This possibility is the most likely, because halothane is known to produce vasodilatation and a decrease in myocardial contractility while maintaining SVR in adults and children.[22]

SaO2% is affected by several factors in patients with cyanotic congenital heart disease: (i) The inspired fraction of O2 (FIO2); (ii) The extent of Rt to Lt shunting; (iii) O2 consumption; (iv) Cardiac output; and (v) The mixed venous oxygen tension.[23] If the magnitude of Rt to Lt shunt is 35% or less, SaO2% would increase when the FIO2 is increased. In our study, SaO2% increased in all groups when the FIO2 increased from 21% (room air) with preoxygenation. Although these increased in SaO2%, we were not able to conclude that Rt to Lt shunt did not increase with any of these anesthetic agents. It should be considered that if the degree of Rt to Lt shunt increased during induction of anesthesia, this was offset by a concomitant decrease in oxygen consumption and increase in cardiac output, both of which increased mixed venous O2 saturation of the shunted blood.[24]

There are some limitations and defects in our study including unrecorded blood pressure of patients during study period and possible inaccuracies or errors in SaO2% values. Some events such as hypothermia, low cardiac output, drug induced vasoconstriction, motion artifact (more common with finger probe than ear or forehead probes monitoring) and sever hypoxia may cause bias or imprecision in SaO2% values. We used finger probe pulse oximetry while movement of patients was inevitable in children especially at the beginning of anesthesia induction.

In conclusion, our study indicated that the use of the IV or IM ketamine and halothane as anesthetic induction agents did not significantly affect SaO2% and all were hinge to especial attention regarding hemodynamic stability.
